# refMLST: reference-based multilocus sequence typing enables universal bacterial typing

**DOI:** 10.1186/s12859-024-05913-4

**Published:** 2024-08-27

**Authors:** Mondher Khdhiri, Ella Thomas, Chanel de Smet, Priyanka Chandar, Induja Chandrakumar, Jean M. Davidson, Paul Anderson, Samuel D. Chorlton

**Affiliations:** 1 BugSeq Bioinformatics Inc, Vancouver, BC Canada; 2 California Polytechnic State University, San Luis Obispo, CA USA

**Keywords:** Multilocus sequence typing, Reference genome, Genomic, Epidemiology

## Abstract

**Background:**

Commonly used approaches for genomic investigation of bacterial outbreaks, including SNP and gene-by-gene approaches, are limited by the requirement for background genomes and curated allele schemes, respectively. As a result, they only work on a select subset of known organisms, and fail on novel or less studied pathogens. We introduce refMLST, a gene-by-gene approach using the reference genome of a bacterium to form a scalable, reproducible and robust method to perform outbreak investigation.

**Results:**

When applied to multiple outbreak causing bacteria including 1263 *Salmonella enterica*, 331 *Yersinia enterocolitica* and 6526 *Campylobacter jejuni* genomes, refMLST enabled consistent clustering, improved resolution, and faster processing in comparison to commonly used tools like chewieSnake.

**Conclusions:**

refMLST is a novel multilocus sequence typing approach that is applicable to any bacterial species with a public reference genome, does not require a curated scheme, and automatically accounts for genetic recombination.

*Availability and implementation*: refMLST is freely available for academic use at https://bugseq.com/academic.

## Background

Traditional approaches for detecting and investigating bacterial outbreaks using DNA sequencing are divided into single-nucleotide polymorphism (SNP) and gene-by-gene approaches, the latter comprising core genome multilocus sequence typing (cgMLST) and whole genome multilocus sequence typing (wgMLST). Multiple comparisons have demonstrated that these approaches largely perform equivalently, with different strengths and weaknesses [1–3]. SNP-based approaches are negatively impacted by genetic recombination, where a single evolutionary event may introduce many SNPs. Popular methods for recombination correction, such as ClonalFrameML [4] and Gubbins [5] give different outputs for different sets of inputs, and therefore cannot be applied sample-by-sample in real-time investigation of a growing outbreak. Furthermore, popular SNP-based approaches only examine nucleotide sites shared across all isolates; as the outbreak grows and genomic diversity increases, fewer sites are conserved across genomes and analysis resolution paradoxically decreases [6, 7]. Conversely, gene-by-gene approaches are limited by the need for a stable, curated, centrally-hosted scheme, limiting their utility to previously studied pathogens [8–10]. Both approaches may be hampered by computational requirements and speed of analysis precluding application to large datasets.

Recently, hashing of locus sequences has been introduced for gene-by-gene approaches to enable robust reproducibility without the need for curating every previously seen allele of a locus in the centrally-hosted scheme [11, 12]. Instead of naming alleles of a locus in the order in which they were discovered, hash-based methods use information theory techniques to identify an allele based on the allele’s sequence. However, these approaches still require a centrally-hosted scheme to define loci locations for hashing, and do not address other aforementioned issues for gene-by-gene approaches. To date, no tool has combined the universal applicability of a reference genome with gene-by-gene analysis, nor enabled recombination-corrected, species-agnostic bacterial outbreak investigation. We present reference-based MSLT (refMLST), a reproducible gene-by-gene approach to type any bacterium based on a single reference genome. refMLST has been tested and validated against different outbreak causing bacteria including *Salmonella enterica*, *Yersinia enterocolitica*, and *Campylobacter jejuni*.

## Implementation

**Fig. 1:**
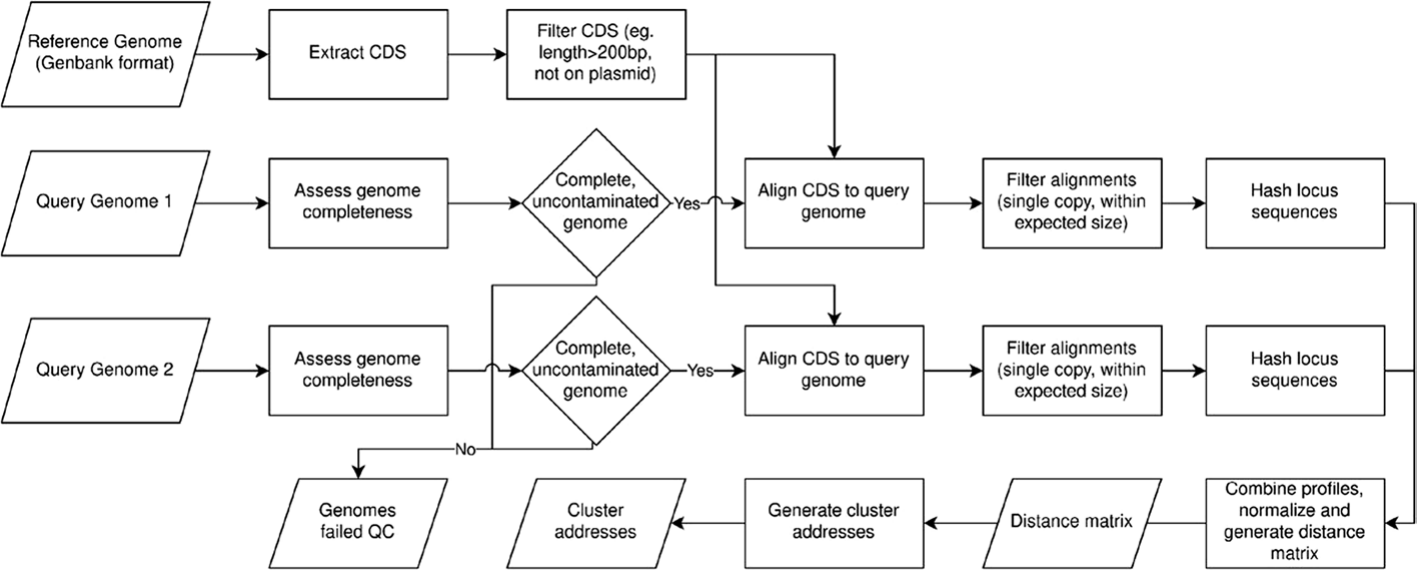
Overview of refMLST workflow. The input/outputs are represented by parallelograms while rectangles reflect processes and diamonds represent decision points

Below, we detail the various steps required for the implementation of refMLST from reference loci identification, typing against a query genome, and combining typing data across genomes to identify outbreak clusters. An overview of this process is provided in (Fig. 1). The query genome for refMLST is a genome assembly in FASTA format which may be generated from whole genome or metagenomic sequencing; however, refMLST requires that the genome is high quality (e.g. if a mixed isolate was sequenced, imperfect taxonomic binning, or strain duplication). refMLST automatically runs BUSCO (v5.3.2) on query genomes to assess completeness and contamination, and will only process those with greater than 90% unfragmented single copy orthologs in accordance with MIMAG standards [13, 14]. All other genomes are flagged for review.

Unlike multilocus sequence typing, cgMLST and wgMLST, refMLST does not require a curated scheme of alleles—it only requires a single reference genome for the pathogen under study, facilitating analysis of any bacterial species. The reference genome in Genbank [15] format is used to perform locus (CDS) identification and extraction from the query genome; this file may be sourced from NCBI for any available genome. For novel pathogens or for use of a reference genome with greater sequence homology to outbreak strains, this file can be annotated from sequenced reference (any sample from outbreaks) using standard genome annotation pipelines. The reference file is parsed to extract coding sequences (CDS), where each CDS represents a reference locus. While reference genomes of closely related species could be used with refMLST if no reference is available for the same species, we limit our analysis below to the use of reference genomes within the same species, preserving the number of CDS shared between query and reference genomes.

Loci on mobile genetic elements may have a different evolutionary history from the bacterial chromosome, comprise a significant component of the accessory genome, contribute to reference bias, and ultimately confound analysis; they are therefore excluded from analysis based on annotation in the Genbank file. As outbreaks of antimicrobial resistance or other important phenotypes may be caused by plasmids, users are encouraged to combine the results of refMLST for host bacterial typing with dedicated plasmid typing tools for a holistic investigation of outbreaks [16]. Additionally, refMLST omits loci less than 200 base pairs in length in accordance with other gene-by-gene analysis tools [17].

To call alleles in the assembled genome of interest, locus sequences from above are rapidly aligned against the query genome assembly using minimap2 (v2.24) with the following command [18]:

minimap2 -a --MD -N 1 --end-bonus 5 query_assembly.fna cds_sequences.fna

Minimap2 can align nucleic acid sequences with up to 20% sequence divergence, enabling identification of genes beyond the expected species boundary of 95% absolute nucleotide identity. We opted not to use protein aligners such as DIAMOND [19] or MMSeqs2 [20] as the former requires the query assembly to be translated into amino acid sequence, while the latter cannot account for frameshifts. Alignments of loci against the genome assembly are filtered for multiple hits to exclude paralogous loci. To ensure quality locus alignments, reference loci must align end to end, and newly discovered alleles must be within 20% of the size of the reference allele. Next, a CRC32 hash [21] is taken of the allele sequence in the query genome to convert it into a compact, easy to understand number.

Evolutionary allele distances between isolates are calculated with a Hamming distance, ignoring missing alleles. The aforementioned genome quality control ensures that small inter-genomic distances are not a result of missing loci in either genome when performing pairwise comparisons.

Cluster addresses are calculated by iterating through query genomes in the order at which they were sequenced/submitted, performing single-linkage hierarchical clustering. Reproducible cluster addresses reflecting inter-sample allele distances are generated; description of similar cluster addresses has previously been published [11, 22]. In brief, cluster addresses are composed of seven digits (“allele addresses”), where each digit reflects a distance threshold (1000, 200, 100, 50, 20, 10, 5 alleles). Addresses are read from left to right, and genomes with greater genomic similarity will share more cluster address digits in common. An example of cluster addresses and their utility is provided in (Table 1). Unlike other address generation mechanisms, and because typing of each genome is deterministic with refMLST, cluster addresses are deterministic and stable, and therefore do not need matching across real-time analyses as an outbreak grows [11, 23]. All samples are output in a distance matrix and line list with cluster codes.

**Table 1:** Example of refMLST cluster address output

Sample name	Merged cluster address	1000 allele address	200 allele address	100 allele address	50 allele address	20 allele address	10 allele address	5 allele address
Reference genome	1.1.1.1.1.1.1	1	1	1	1	1	1	1
Sample 1	2.1.1.1.1.1.1	2	1	1	1	1	1	1
Sample 2	2.1.1.1.1.2.1	2	1	1	1	1	2	1
Sample 3	2.1.1.1.1.2.2	2	1	1	1	1	2	2

The reference genome is always assigned the first cluster, 1.1.1.1.1.1.1. Sample 1 is over 1000 alleles from the reference genome, as the first digit (1000 allele address) is different between these two cluster addresses. Sample 2 is more than 10 but less than 20 alleles different from sample 1, as the 10 allele address is different. Sample 3 is more than 5 but less than 10 alleles different from sample 2, and more than 10 but less than 20 alleles different from sample 1. Cluster addresses do not change over time as genomes are added to the outbreak analysis

To assess refMLST performance against other gene-by-gene approaches, namely the cgMLST and the wgMLST, we applied refMLST on data collected from different bacterial outbreaks caused by *S. enterica* [11], *Y. enterocolitica* [24], and *C. jejuni* [25]. For *Y. enterocolitica*, and *C. jejuni*, genomic data for 331 and 6526 isolates, respectively, were recovered from public databases with their corresponding whole and core allelic schemes [24, 25]. For *S. enterica*, 1263 isolates with publicly available sequencing data were collected with their corresponding core allelic schema for the cgMLST analysis and results were compared to those generated by chewieSnake (v3.0.0), a recently published, hash-based cgMLST tool [11]. Meanwhile, for the wgMLST based comparison, genome assemblies recovered from [11] were processed through the chewBBACA (v3.3.0) standard workflow to generate the required whole genome schema and the allelic profile. Briefly, all the 1263 genomes were used to create the schema and the allele call using the chewBBACA CreateSchema.py script with default settings. The quality of the loci has been assessed using the schema evaluation script from the chewBBACA suit and paralogous genes were removed alongside loci with single alleles. Cgmlst-dists (v0.4.0) has been used to generate the final wgMLST distance matrix [26].

## Results

refMLST does not require a curated scheme of alleles—it only requires a single reference genome, facilitating analysis of any bacterial species. refMLST reduces bias from recombination by examining whole genes instead of individual SNPs, reducing the need for post-analysis, non-deterministic recombination correction. Consequently, cluster addresses are reproducible when generated from the same input assemblies, and therefore do not need matching across real-time analyses as an outbreak grows [11, 23].

**Fig. 2:**
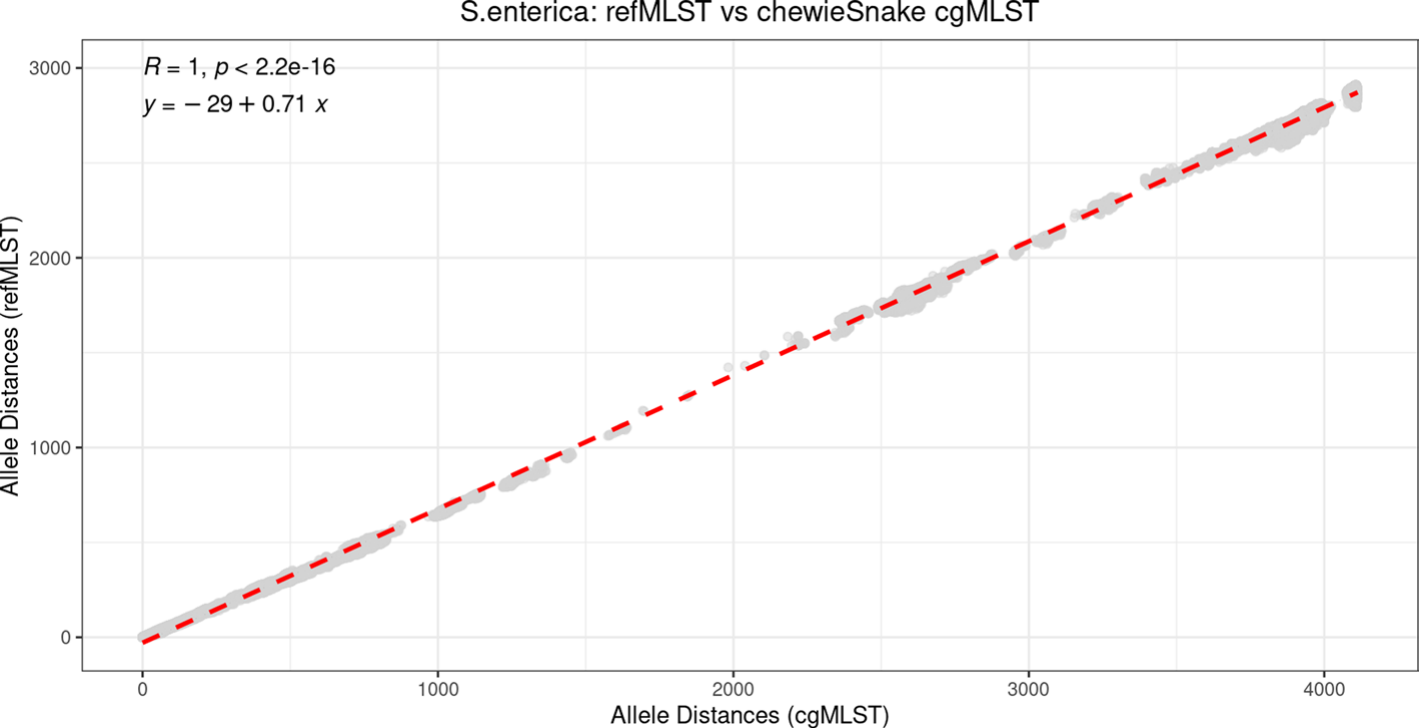
refMLST pairwise allele distances compared with chewieSnake pairwise allele distances for 1263 *Salmonella enterica* genomes. Each dot represents a comparison of two genome distances

**Fig. 3:**
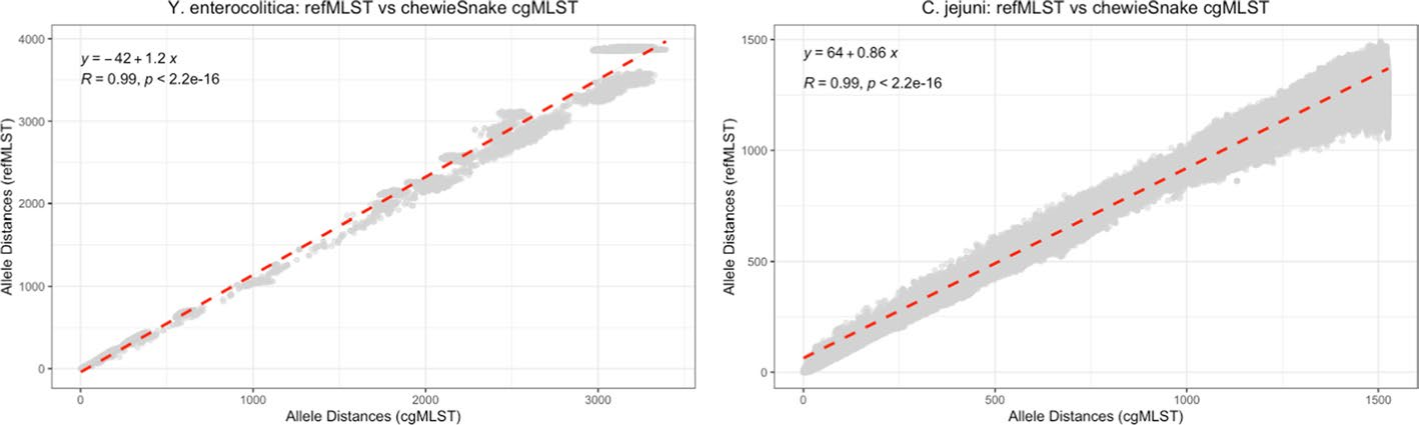
refMLST pairwise allele distances compared with chewieSnake pairwise allele distances for 331 *Yersinia enterocolitica* genomes and 6526 *Campylobacter jejuni* genomes. Each dot represents a comparison of two genome allele distances and the overall linear regression and R value are displayed

**Fig. 4:**
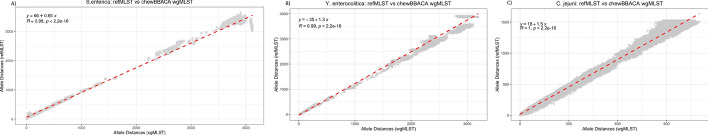
refMLST pairwise allele distances compared with chewBBACA wgMLST pairwise allele distances for **A** 1263 *Salmonella enterica*, **B** 331 *Yersinia enterocolitica* and **C** 6526 *Campylobacter jejuni* genomes. Each dot represents a comparison of two genome allele distances and the overall linear regression and R value are displayed

When results obtained using refMLST were compared with cgMLST and wgMLST, the other two popular gene-by-gene approaches, a very high level of concordance was reported (Figs. 2, 3 and 4). When cgMLST was applied to all tested bacterial species, we found that the spearman correlation between the refMLST and the chewieSnake allele distances is 0.98 for *Y. enterocolitica*, and 0.99 for both *S. enterica* and *C. jejuni* (Table 3). In the case of *S. enterica* (Fig. 2) and at distances less than 100 refMLST alleles, refMLST distances are on average 20 (SD: 10) alleles greater than chewieSnake, indicating an enhanced ability to resolve closely related isolates. The increased allele distances are explained by the examination of a greater number of loci when comparing genomes (refMLST: 4271 loci, chewieSnake: 3000 loci). Accounting for differences in allele distances between tools with linear regression, we find outbreak clusters at comparable allele distances (refMLST: 20 alleles, chewieSnake: 10 alleles) to be highly correlated with an adjusted rand index (ARI) of 0.92 [27]. refMLST is also rapid: refMLST processed the 1263 *S. enterica* genomes in 59 min on an r6i.2xl EC2 instance with 8 CPUs and 64 GB of RAM; in contrast, chewieSnake took 23 h and 33 min with the same data and server (Table 2). Similar trends were also obtained when refMLST was compared with results from the wgMLST based approaches (Fig. 4) with the lowest spearman correlation value of 0.91 for *Y. enterocolitica* and the highest being 0.99 for both *S. enterica* and *C. jejuni* (Table 3).

**Table 2:** Comparison of time efficiency data between refMLST, chewBBACA and chewieSnake for *S. Enterica*, *Y. Enterocolitica*, and *C. Jejuni* on a Linux machine with 100 GB of RAM and 10 CPUs

	refMLST	chewBBACA	chewieSnake	Number of processed genomes
*S. enterica*	0.98 h*	NA	23.55 h*	1263
*Y. enterocolitica*	0.22 h	2.72 h	46.8 h	331
*C. jejuni*	6.23 h	9.49 h	166.5 h	6526

*The analysis was performed using an r6i.2xl EC2 instance with 8 CPUs and 64 GB of RAM

**Table 3:** Comparison of spearman correlation coefficient between refMLST allele distances and distances computed based on cgMLST and wgMLST approaches

	cg/wgMLST based approaches					
	* S. enterica *		* Y. enterocolitica *		* C. jejuni *	
	cgMLST	wgMLST	cgMLST	wgMLST	cgMLST	wgMLST
refMLST	0.99	0.99	0.99	0.91	0.98	0.99

## Discussion

Here, we present a rapid and accurate method for gene-by-gene outbreak analysis of any bacterial species. Given predictions of millions to trillions of microbial species on earth, it is infeasible to develop cgMLST or wgMLST schemes for each species [28–30]. Even limiting analysis to bacterial species infecting humans yields a prohibitive number of species for cg/wgMLST analysis, with 1513 species known to infect humans and an exponential growth in the discovery of new species infecting humans [30]. Furthermore, 27% of these species have infected fewer than 3 humans [30]. Methods are needed which are universally applicable for prokaryotes and do not require a large number of genomes to initiate outbreak surveillance. refMLST leverages the annotation of a single prokaryotic genome to initiate a gene-by-gene analysis, and can therefore be applied early in the onset of an outbreak or in outbreaks with limited numbers of cases. As the initial genome annotation is stable, additional genomes can be added to an analysis to generate stable cluster addresses as surveillance efforts grow.

refMLST has several limitations. First, a seed annotation is required to perform analysis; the similarity of the seed annotation to query genomes may impact the number of loci in each pairwise comparison. We leverage the NCBI annotations, as NCBI’s annotations are freely available for all genomes on RefSeq and the PGAP pipeline has been curated and validated [31]. While we have not evaluated refMLST with tools such as Prokka or Bakta, we do not anticipate issues; for example, Bakta follows NCBI’s specifications [32, 33]. Second, refMLST leverages minimap2 for finding loci from the query annotation in the target genome. As minimap2 is designed for nucleotide alignment, it is conceivable that highly divergent or fragmented loci between the reference and target genome will be missed. We and others have previously shown that minimap2 performs well at 85% absolute nucleotide identity (ANI) and above, which is well below the bacterial species demarcation of 95%, and should therefore be suitable provided the annotation derives from the same bacterial species as the target [17, 34, 35].

We ultimately chose minimap2 [18] as our sequence aligner in refMLST as it enables rapid analysis and is robust to frameshifts which may affect protein alignment methods. Here we have demonstrated that refMLST can easily scale to thousands of genomes across different branches of the bacterial taxonomy with only hours of analysis. The speed of this analysis, without the need to curate schemes or perform recombination correction, enables the real-time surveillance, detection and investigation of bacterial outbreaks.

## Conclusion

With the growing global burden of disease attributable to bacterial outbreaks, including foodborne and hospital-acquired infections, novel methods for detection and investigation of bacterial outbreaks via genomics are needed. refMLST does not require background genomes as for SNP-based approaches, and we demonstrate its application to important human pathogens including *S. enterica*, *Y. enterocolitica* and *C. jejuni*. refMLST combines the advantages of SNP and gene-by-gene approaches to enable reproducible, universal bacterial typing without suffering from each approach’s limitations.

## Availability and requirements

Project name: refMLST.

Project home page: https://bugseq.com/academic.

Operating system(s): Web browser.

Programming language: Python.

Other requirements: None.

License: refMLST is freely available for academic use at https://bugseq.com/academic.

Any restrictions to use by non-academics: Contact the corresponding author for a license.

## Data Availability

*Salmonella enterica* genomes are available from [11]. *Yersinia enterocolitica* genomes availabl*e* from [24]. *Campylobacter jejuni* genomes available from [25]. refMLST is freely available for academic use at https://bugseq.com/academic. Documentation on refMLST, including input requirements and interpretation of outputs is available at https://docs.bugseq.com.

## Abbreviations

ANI: Absolute nucleotide identity
CDS: Coding sequences
cgMLST: Core genome multilocus sequence typing
MIMAG: Minimum information about metagenome-assembled genome
PGAP: Prokaryotic genome annotation pipeline
refMLST: Reference multilocus sequence typing
SNP: Single nucleotide polymorphism
wgMLST: Whole genome multilocus sequence typing
